# Chronic brucellosis presenting as cardiac implantable electronic device–related endocarditis: diagnostic challenges and management—a case report

**DOI:** 10.1093/ehjcr/ytag540

**Published:** 2026-07-21

**Authors:** Istiklal Ozkaya, Taner Ulus, Emine Gokcen Kuru, Emre Entok

**Affiliations:** Department of Cardiology, Faculty of Medicine, Eskisehir Osmangazi University, 26040 Eskisehir, Turkiye; Department of Cardiology, Faculty of Medicine, Eskisehir Osmangazi University, 26040 Eskisehir, Turkiye; Department of Cardiology, Faculty of Medicine, Eskisehir Osmangazi University, 26040 Eskisehir, Turkiye; Nuclear Medicine, Faculty of Medicine, Eskisehir Osmangazi University, 26040 Eskisehir, Turkiye

**Keywords:** Brucellosis, Cardiac implantable electronic device infection, Infective endocarditis, Lead extraction, PET/CT, Case report

## Abstract

**Background:**

Brucellosis is a zoonotic infection that may rarely involve the cardiovascular system, causing infective endocarditis. Diagnosis of Brucella-associated cardiac implantable electronic device (CIED) endocarditis is challenging because of its indolent course and frequently negative blood cultures.

**Case summary:**

A 42-year-old shepherd with an implantable cardioverter–defibrillator had experienced fever, myalgia, arthralgia, and weight loss for approximately 18 months. During this period, investigations for malignancy and rheumatologic diseases were unrevealing. He was subsequently diagnosed with brucellosis at another institution based on a Brucella serum agglutination test positive at a titre of 1:1280, and antibiotic therapy was initiated. Three weeks later, he was referred to our centre because of generator pocket erosion and purulent drainage. Tissue cultures from the pocket and repeat blood cultures showed no growth. Transoesophageal echocardiography demonstrated a 14 × 8 mm mobile mass attached to the right ventricular lead, suspicious for vegetation. Fluorine-18 fluorodeoxyglucose positron emission tomography/computed tomography (^18^F-FDG PET/CT) demonstrated increased metabolic uptake around the lead. According to the diagnostic criteria of the 2023 European Society of Cardiology Endocarditis Guidelines, Brucella CIED-related endocarditis was diagnosed. Antimicrobial therapy was revised, followed by complete percutaneous device extraction. The patient recovered without recurrence, and device re-implantation was not indicated.

**Discussion:**

Brucella infection should be considered in patients from endemic regions or high-risk occupational groups presenting with culture-negative CIED infection. In such cases, serological testing combined with transoesophageal echocardiography and ^18^F-FDG PET/CT is essential for diagnosis. Complete device extraction with targeted antimicrobial therapy is crucial, and re-implantation should be individualized.

Learning points

- In culture-negative cardiac implantable electronic device (CIED) infection, infective endocarditis should not be overlooked; serology and multimodality imaging are key to establishing the diagnosis.
- Complete device extraction and targeted antimicrobial therapy are the cornerstone of treatment; the indication for device re-implantation should be carefully reassessed.

## Introduction

Brucellosis is a zoonotic disease with a broad clinical spectrum and may present with organ involvement, recurrent attacks, and treatment-resistant courses. More than half of patients demonstrate involvement of various organ systems, including the cardiovascular system, among which endocarditis represents the most frequent and fatal manifestation, occurring in approximately 1% of cases.^1,2^

In recent years, the increasing use of cardiac pacemakers and defibrillators has been accompanied by a parallel rise in cardiac implantable electronic device (CIED) infections. Although Brucella endocarditis accounts for a small proportion of these infections, it is clinically significant due to its non-specific presentation, frequent culture negativity, and diagnostic challenges, often leading to delayed diagnosis. Here, we present a case of Brucella-related CIED endocarditis diagnosed approximately 1.5 years after symptom onset and discuss diagnostic and therapeutic considerations in light of current evidence.

## Summary figure

CIED: Cardiac implantable electronic device, CRP: C-reactive protein, ESC: European Society of Cardiology, ESR: Erythrocyte sedimentation rate, FDG PET/CT: fluorodeoxyglucose positron emission tomography/computed tomography, ICD: Implantable cardioverter-defibrillator, LGE: Late gadolinium enhancement, LVEF: Left ventricular ejection fraction, MR: magnetic resonance RV: Right ventricle, SAT: standard agglutination test, TEE: Transoesophageal echocardiography, TTE: Transthoracic echocardiography.

**Figure:**
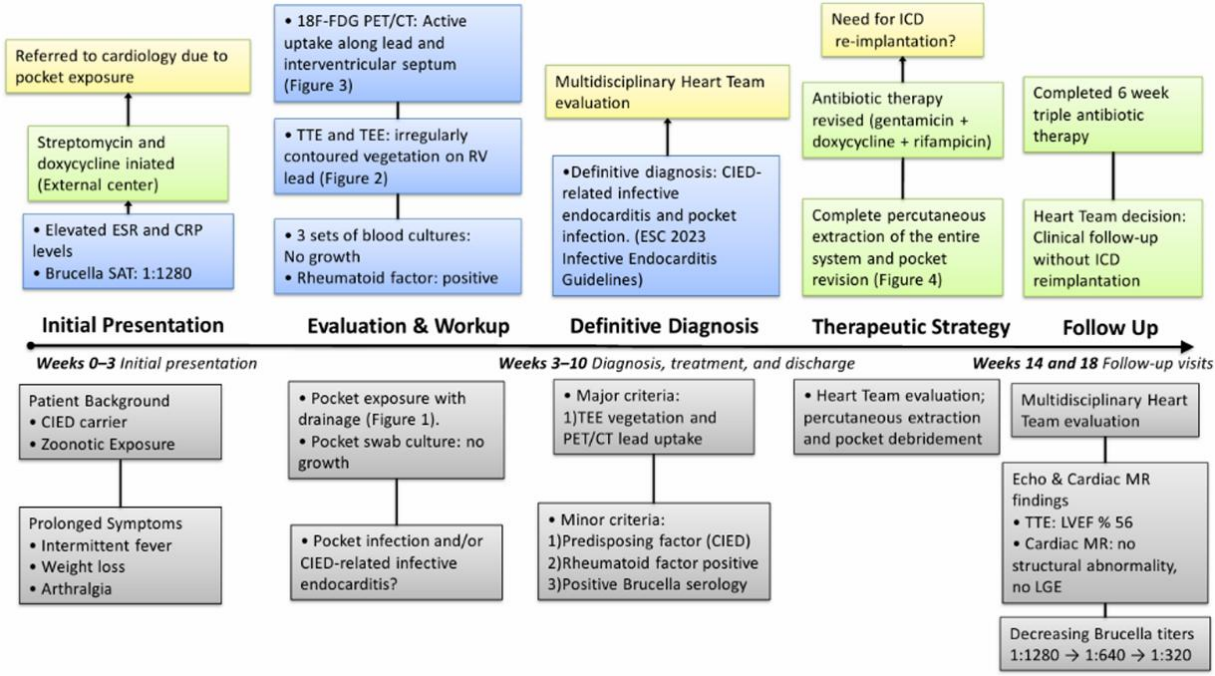


## Case presentation

A 42-year-old shepherd had undergone dual-chamber implantable cardioverter–defibrillator (DDD-ICD) implantation for a suspected cardiac arrest at an external centre in 2015. Coronary ischaemia was excluded, and transthoracic echocardiography (TTE) demonstrated a left ventricular ejection fraction (LVEF) of 38%. TTE also demonstrated deep LV trabeculations. Possible LV non-compaction cardiomyopathy was suspected. An ICD was implanted for secondary prevention. Cardiac magnetic resonance imaging (MRI) was not performed before implantation. The patient reported intermittent fever, chills, and unintentional weight loss for 18 months without a definitive diagnosis. Repeated investigations, including thoracoabdominal computed tomography (CT), excluded malignancy. Progressive arthralgia, myalgia, and gait difficulty prompted rheumatologic evaluation. Laboratory studies demonstrated elevated erythrocyte sedimentation rate and C-reactive protein levels. Because of suspected zoonotic infection, Brucella tube agglutination testing (TAT) and the Brucella Coombs gel test (BCGT) were both positive at a titre of 1:1280. Streptomycin–doxycycline therapy was initiated.

**Figure 1:**
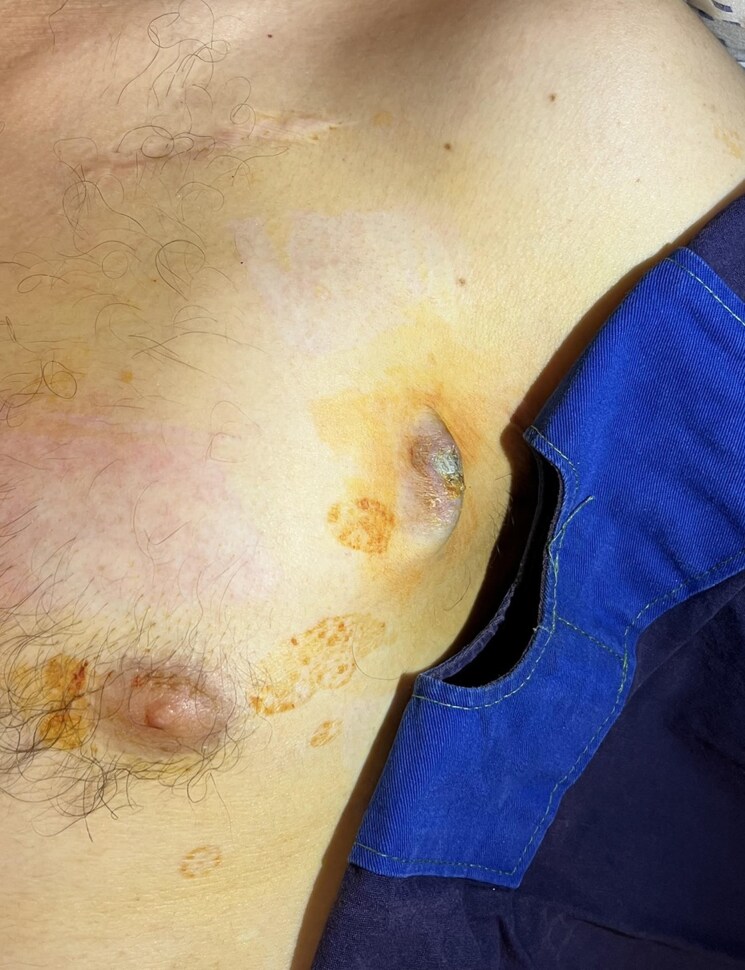
The patient's exposed generator area.

**Figure 2:**
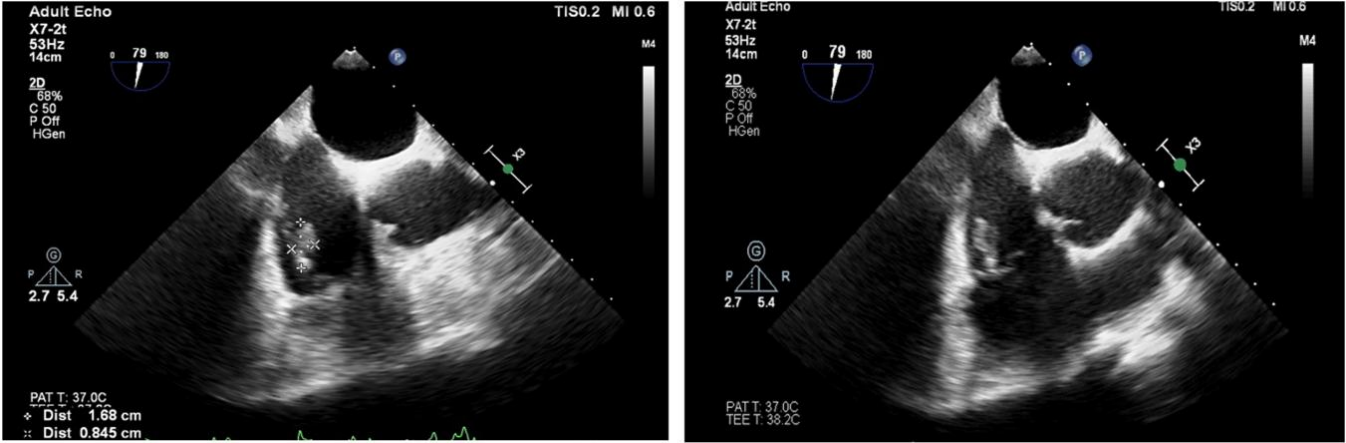
Transoesophageal echocardiography images of the patient. White arrows indicate a mass suspicious for vegetation at the lead tip.

During the third week of antibiotic therapy, the patient presented with generator exposure and drainage at the pacemaker pocket site (*Figure 1*). Swab cultures obtained from the pocket were negative. To evaluate suspected CIED-related infective endocarditis, both TTE and transoesophageal echocardiography (TEE) were performed. TEE revealed a mobile, hypoechoic, irregular 14 × 8 mm mass attached to the right ventricular lead, suspicious for vegetation (*Figure 2*).

**Figure 3:**
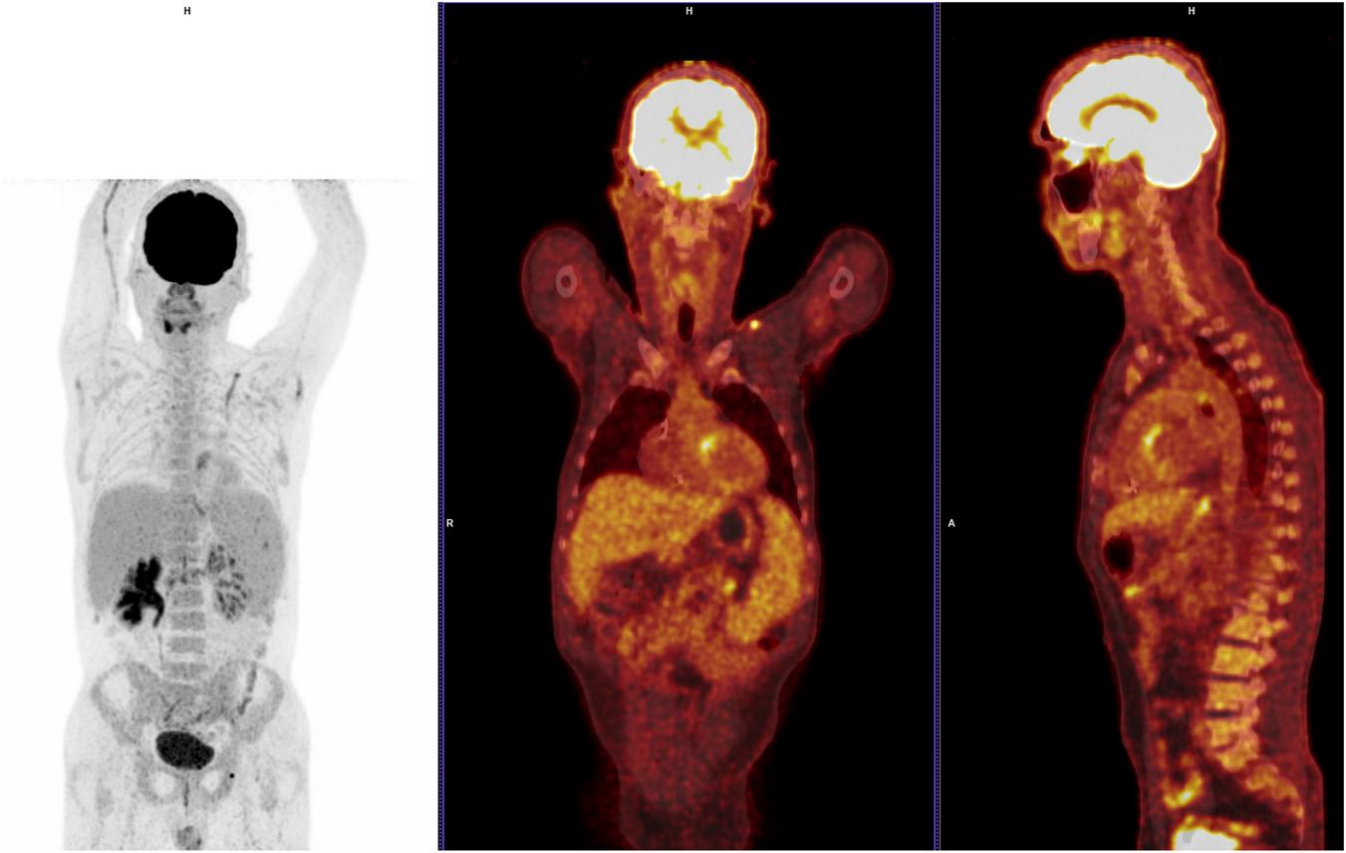
Cardiac implantable electronic device lead infection visualized by fluorine-18 fluorodeoxyglucose positron emission tomography/computed tomography. (*A*) Coronal positron emission tomography images. (*B*) Positron emission tomography fusion images.

^18^F-fluorodeoxyglucose positron emission tomography/CT (^18^F-FDG PET/CT) demonstrated increased uptake along the proximal lead and interventricular septum, without evidence of bone or joint involvement (*Figure 3*). Three consecutive sets of blood cultures were negative. The diagnosis of Brucella CIED-related endocarditis was established based on one major criterion (imaging evidence on TEE) and three minor criteria, including a predisposing factor (presence of a CIED), an immunologic phenomenon (positive rheumatoid factor), and significantly positive serology.^3^ Antibiotic therapy was revised to a combination of gentamicin, doxycycline, and rifampicin.

**Figure 4:**
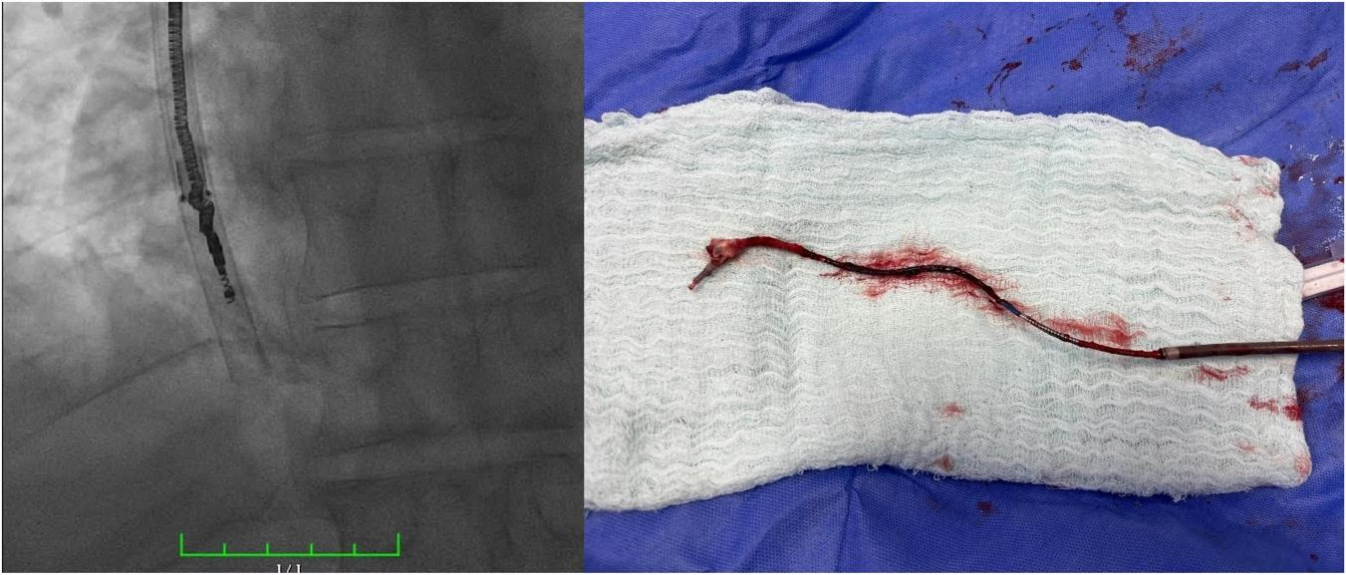
(*A*) Fluoroscopic images of the evolution RL controlled-rotation dilator sheath set and the extracted implantable cardioverter–defibrillator lead. (*B*) Intraoperative image of the extracted implantable cardioverter–defibrillator lead.

Following Heart Team evaluation, open surgical extraction was considered high risk. Therefore, complete percutaneous extraction was planned. With support from the cardiothoracic and plastic-reconstructive surgery teams, the procedure was performed in the cardiac catheterization laboratory under general anaesthesia, with femoral venous access obtained for volume resuscitation and temporary pacing. Extraction was performed via the left subclavian vein. After sterile exposure of the pocket, the generator was removed, and the leads were dissected from surrounding fibrous tissue. Both leads had active fixation mechanisms. Initial attempts with simple traction and a locking stylet (Liberator, Cook Medical, Bloomington, IN, USA) were unsuccessful; therefore, an 11-French rotational mechanical sheath (Evolution, Cook Medical) was used, enabling complete lead removal (*Figure 4*).

Subsequently, necrotic tissue surrounding the generator pocket was debrided. Tissue samples from the extracted leads and pocket were sent for microbiological analysis. The procedure was completed without complications, and post-procedural echocardiography showed mild tricuspid regurgitation, consistent with baseline findings.

Postoperative transthoracic echocardiography showed an LVEF of 56%, and cardiac MRI revealed no structural abnormalities or late gadolinium enhancement. Device interrogation demonstrated no malignant ventricular arrhythmias or appropriate ICD therapies. Based on these findings, the Heart Team decided against re-implantation of the device and opted for clinical follow-up.

Triple antibiotic therapy was completed over six weeks and discontinued. Serial Brucella agglutination titres decreased from 1:640 to 1:320, and the patient remained clinically stable. He was discharged without device implantation and remained asymptomatic at the 1-month outpatient follow-up.

## Discussion

We present a case of CIED-related Brucella endocarditis in which the entire device system was successfully removed by complete lead extraction. This case is noteworthy for several reasons. First, brucellosis may be diagnosed late because of its non-specific clinical presentation and frequent negative blood cultures, even in active disease; in patients with CIEDs, lead endocarditis may represent the initial manifestation and should be considered in endemic regions, particularly in patients with occupational exposure and prolonged fever and weight loss. Second, in culture-negative cases, serological testing combined with adjunctive imaging modalities, including PET/CT, is essential for confirming active infection. Third, dense adhesions may form around Brucella-infected vegetations; therefore, when percutaneous extraction is planned, the availability of appropriate extraction tools, such as locking stylets and rotational mechanical sheaths, is crucial. Finally, following complete system removal, the indication for device re-implantation should be reassessed by a multidisciplinary Heart Team. In our case, no bacterial growth was detected in blood or lead tissue cultures. This finding can be explained by two factors: prior antibiotic therapy for 3 weeks before presentation and the well-documented culture negativity observed in a substantial proportion of active Brucella infections.

Elzein *et al*. reported culture negativity in 40% of patients in their case series.^4^ Serum agglutination testing remains a cornerstone of Brucella diagnosis in culture-negative cases. In our case, serological testing included TAT and BCGT, with titres ≥1:160 considered positive for both tests. At this cut-off, the reported sensitivity and specificity of TAT are 71.4% and 100%, respectively, compared with 78.8% and 93.8% for BCGT. Enzyme-linked immunosorbent assay can also be used to diagnose brucellosis. However, no single serological test is sufficient; combining serological methods improves diagnostic accuracy.^5,6^ Positive titres should be interpreted together with clinical and imaging findings, as seropositivity may persist after previous infection.^7^

In addition, ^18^F-FDG PET/CT imaging demonstrated increased metabolic uptake along the lead, supporting the diagnosis of CIED-related infection. PET/CT has been shown to improve diagnostic sensitivity in device-related infections and may identify extracardiac infectious foci.^4^ The 2024 ACC Expert Consensus Document also recommends ^18^F-FDG PET/CT as a valuable adjunct in cases with inconclusive echocardiography, negative cultures, or suspected lead or pocket infection.^8^

Brucella infective endocarditis should be treated with at least 4 weeks of prolonged combination antibiotic therapy consisting of doxycycline and rifampicin, with consideration of adjunctive gentamicin during the initial weeks of treatment.^3,9^ In cases of definite endocarditis with clear CIED involvement, as in our case, extraction of the entire device system is recommended at a Class I recommendation level.^3^ In our patient, treatment with rifampicin, doxycycline, and gentamicin resulted in rapid clinical improvement after complete system extraction, with close serological monitoring used to assess treatment response.^10^

Finally, reassessment of the indication for CIED re-implantation is essential after system removal.^8^ In our patient, the initial history of cardiac arrest was uncertain, and follow-up echocardiography and cardiac MRI showed no structural heart disease; therefore, ICD re-implantation was unnecessary.

Histopathological examination and direct microscopic analysis of the extracted material could not be performed because the material was no longer available after microbiological culture processing.

## Conclusion

Brucella endocarditis should be considered in patients with relevant epidemiological or occupational risk factors. In culture-negative cases, careful interpretation of serological findings combined with multimodality imaging, including TTE, TEE, and PET/CT, is essential for accurate diagnosis. Early and complete removal of the infected device system, together with appropriately targeted antimicrobial therapy, is crucial for reducing mortality. Decisions regarding device re-implantation should be individualized and made after thorough reassessment following eradication of the infection.

## Data Availability

The data underlying this article are available within the article. Additional data are not publicly available in order to protect patient confidentiality.

## Lead author biography

Istiklal Ozkaya, MD, graduated from Necmettin Erbakan University Faculty of Medicine in 2021. Since 2022, he has been working at the Department of Cardiology, Faculty of Medicine, Eskişehir Osmangazi University, Türkiye. His clinical and research interests focus on cardiac arrhythmias, cardiac implantable electronic devices (CIEDs), and cardiovascular imaging.
